# Blast Nucleation Suppressed Growth of Large-Sized High-Quality CsPbBr_3_ Single Crystals for Photodetector Applications

**DOI:** 10.3390/molecules30224423

**Published:** 2025-11-16

**Authors:** Xinyu Sun, Yuxia Yin, Xiaolin Xia, Teng Zhang

**Affiliations:** 1 Shan Dong Key Laboratory of Intelligent Energy Materials, School of Materials Science and Engineering, China University of Petroleum (East China), Qingdao 266580, China; upc_sxy9764@163.com (X.S.); z23140095@s.upc.edu.cn (Y.Y.); 2 School of Information Science and Engineering, Qingdao Institute of Technology, Qingdao 266300, China; xiaxiaolin@qit.edu.cn

**Keywords:** perovskite photodetector, single crystal, suppressed blast nucleation, inverse temperature crystallization (ITC) method

## Abstract

During the growth of lead halide perovskite single crystals (SCs) with the conventional inverse temperature crystallization (ITC) method, the blast nucleation of the precursor under supersaturation conditions is always unavoidable. In the current study, three kinds of additives namely methanol (MOE), ethyl alcohol (EtOH), and polyethylene glycol (PEG) are introduced to regulate the growth of CsPbBr_3_ SCs. Benefiting from the strong anchoring hydroxy groups (-OH) with the Pb^2+^ species, large-sized CsPbBr_3_ crystals with reduced defect densities were prepared (PEG-regulated). In addition, the viscosity of the precursor solution increases after adding PEG additive, which provides a more stabilized environment for crystal growth. Finally, the photodetectors prepared from our PEG-tuned CsPbBr_3_ SCs show a responsivity of 2.25 A/W and a detectivity of 6.06 × 10^11^ Jones, demonstrating the potential of CsPbBr_3_ SCs for photo-detecting applications.

## 1. Introduction

Recently, lead halide perovskites with ABX_3_ structure are drawing interest due to their optoelectronic applications [1,2,3,4,5]. Lead halide perovskites can be classified as polycrystalline thin films, low-dimensional nanostructures, and single crystals (SCs) [6]. The polycrystalline perovskite thin films are regarded as the ideal choice for high-performance solar cells [1,7,8], while the light-emitting devices are based on perovskite low-dimensional nanostructures [9,10]. As for perovskite SCs with reduced defect densities and extended carrier diffusion length, they are showing great potential as photodetectors, X-ray detectors, and γ-ray detectors [11,12,13,14,15]. Liu and co-workers demonstrated that the photo-detecting performance of the FAPbI_3_ SCs was much better than that of the polycrystalline films [6]. C. Su reported the CH_3_NH_3_PbBr_3_ narrowband photodetector displayed high response under a low bias (−1 V) [16]. An ultraviolet (UV) detector prototype has been demonstrated by M. Bakr using MAPbCl_3_ SCs [17]. Despite progressive achievements, the organic cations (CH_3_NH_3_^+^, FA^+^, CH(NH_2_)_2_^+^), etc.) of the organic–inorganic hybrid perovskite (OIHP) are extremely sensitive to environmental humidity and oxygen, resulting in poor stability [18]. Thus, CsPbBr_3_ SCs with strong chemical bonding stand out and are being extensively investigated [19,20,21]. The stability of selective devices can be further enhanced via encapsulation [22,23].

The performance of the perovskite photodetector is highly dependent on the quality of the synthesized SCs [24]. Thus, an elaborate control of the nucleation and growth of the perovskite SCs turns out to be the first step towards high-performance photodetectors. Generally, the vertical Bridgman method and solution growth method are widely used in the growth of CsPbBr_3_ SCs [25,26,27,28,29]. X. Tao reported centimeter-scale CsPbBr_3_ SCs with the modified Bridgman method [25]. H. Zeng suggested that the defect densities of the Bridgman method-grown CsPbBr_3_ SCs can be as low as 1 × 10^9^ cm^−3^ [26]. Despite the progressive development, the Bridgman method requires high temperature processing and several post-treatments, which increase the difficulty of the as-synthesized SCs. Moreover, phase transition is frequently unavoidable in Bridgman-grown crystals, which brings external defect densities [27].

The solution growth method refers to the nucleation and growth of the precursor in a supersaturation condition. Inverse temperature crystallization (ITC) and anti-solvent vapor crystallization (AVC) methods are frequently used in the solution-grown CsPbBr_3_ SCs. With methanol as the anti-solvent, X. Zhan revealed the 2D nucleation growth of the CsPbBr_3_ SCs [30]. W. Jie prepared centimeter-scale CsPbBr_3_ SCs with the AVC method and demonstrated effective light detection in a wavelength region of 365–420 nm [31]. In 2016, M.V. Kovalenko demonstrated that the ITC method can be used for growing CsPbBr_3_ SCs [30]. Different from the Bridgman method, the solution-grown method can be processed in ambient conditions with a temperature of less than 150 °C, which is advantageous for low-cost production. However, the ITC-grown SCs frequently suffer from tiny crystals with irregular shapes. This is mainly due to the blast nucleation of the precursors right-reaching the steep part of the solubility curve [30]. Thus, the controlled release of the solute has become the key issue in the ITC-processed perovskite SCs. M. V. Kovalenko suggested that a mixed solvent was essential for the ITC-grown CsPbBr_3_ and the growth can be controlled via changing temperature [30]. CyOH has been introduced to reduce the secondary phase (SP) defects in the ITC-grown CsPbBr_3_ SCs [31]. Y. Feng demonstrated that the choline bromide (CB) can be used to regulate the growth and the exposed facet of the synthesized CsPbBr_3_ SCs [32]. X. Zhao further proved the importance of CB in the growth of CsPbBr_3_ SCs [20]. X. Han proposed that polymer additives with oxygen groups are effective regulators during the growth of large-sized compositional varied perovskite SCs. It seems that additive engineering is an effective strategy during the synthesis of high-quality large-sized CsPbBr_3_ SCs.

In this work, we introduce 2-Methoxyethanol (MOE), anhydrous ethanol (EtOH), and polyethylene glycol (PEG) as additives to tune the growth of CsPbBr_3_ SCs. The oxygen-containing additives bond strongly with the lead precursors and contribute to the gradual precipitation of the solute, and irregularly shaped crystals caused by the blast nucleation of the precursors are eliminated. With PEG additives, the largest crystal size of 6 mm × 3 mm × 1 mm with reduced defect densities has been achieved. As for device performance, the photodetectors prepared from our PEG-assisted SCs show a responsivity of 2.25 A/W and a detectivity of up to 6.06 × 10^11^ Jones, showing their advantage for light-detection applications.

## 2. Results and Discussion

**Figure 1:**
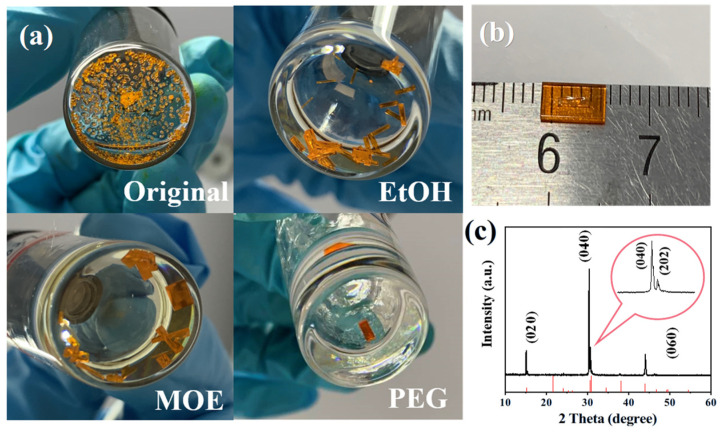
(**a**) Photographs of different additive-regulated CsPbBr_3_ SCs. (**b**) Photographs of the best PEG-regulated CsPbBr_3_ SCs. (**c**) Single-crystal X-ray diffraction analysis of the as-synthesized CsPbBr_3_ SCs.

First, DMSO has been selected as the solvent of ITC-grown CsPbBr_3_ SCs. The molar ratio of CsBr and PbBr_2_ has been fixed to 1:2 to avoid Cs_4_PbBr_6_ by-product formation [33]. Shown in Figure 1a, although the seed-assisted method has been used, we receive a vast number of tiny crystals after the evaporation of the solvent. While large-sized crystals can be obtained from the additive-regulated ITC solutions. It seems that the alcohol-based additives can, to a great extent, suppress the problem of blast nucleation during CsPbBr_3_ SC growth. The PEG additive turns out to be the most effective, resulting in large-sized single crystals. The concentration of the PEG additive has also been investigated in detail (Figure S1). Although both contribute to large crystals, the surface of 0.05 g PEG-regulated SCs shows visible pits, so 0.1 g of additive has been used in our follow-up studies. Using PEG as additive, we receive a maximum-sized CsPbBr_3_ SC with a length of 6 mm (Figure 1b). More photographs of the as-synthesized crystals are summarized in Figure S2.

According to Figure 1c, the synthesized CsPbBr_3_ SCs can be indexed to an orthogonal phase (PDF#54-0751) [34]. The morphological characterizations of the as-synthesized SCs are listed in Figures S3 and S4. As shown in the scanning electron microscope (SEM) images, a step-like structure has been detected in the control SCs, indicating the poor quality of the control SCs. Pinholes and cracks are clear in crystals with EtOH and MOE additives. Only the PEG additive results in a smooth surface with fewer defects, which is beneficial for optoelectronic applications. The high quality of the PEG-regulated SCs is also supported by atomic force microscope (AFM) characterization (Figure S4).

**Figure 2:**
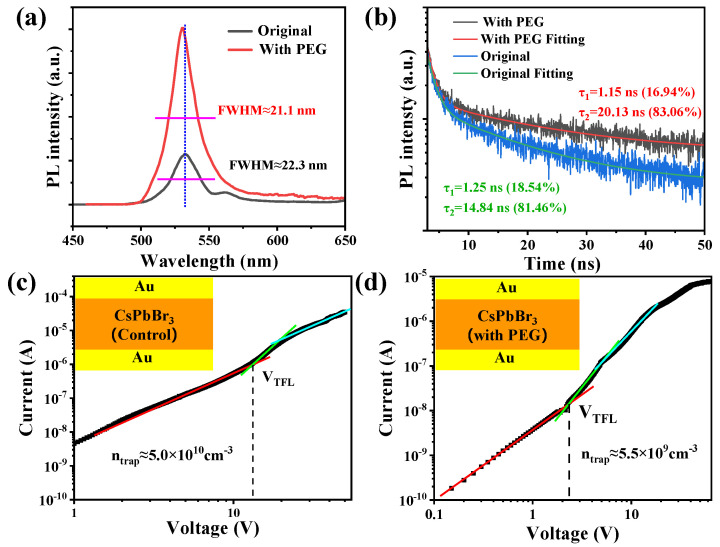
(**a**) Steady—state photoluminescence (PL) and (**b**) transient PL (TRPL) spectrum of the control and PEG—regulated CsPbBr3 SCs. Current—voltage (J—V) characteristics of the control (**c**) and PEG—regulated (**d**) CsPbBr_3_ SCs.

Compared with the control SCs, PL intensity has been dramatically enhanced in PEG-regulated ones (Figure 2a). In addition, a relatively lower full width at half maxima (FWHM) of the PL spectrum has been detected in PEG-regulated SCs. Moreover, the sub-peak at ~560 nm of the control SCs disappeared in the PEG-regulated samples, indicating a reduced defect density. The TRPL characterization has been listed in Figure 2b. The TRPL curves follow a bi-exponential decay fitting. The fast time component (*τ*_1_) can be regarded as surface recombination and the slow time component (*τ*_2_) is bulk recombination. The average PL lifetime (*τ_ave_*) has increased from 14.58 ns to 20.13 ns, and can be calculated via the following equation:

(1) $$\;\tau_{ave}=\frac{A_{1}\tau_{1}^{2}\;+\;A_{2}\tau_{2}^{2}}{A_{1}\tau_{1}\;+\;A_{2}\tau_{2}}$$

The above PL characterization supports the improved quality of the PEG-regulated CsPbBr_3_ SCs. With the assistance of the space charge limiting current (SCLC) analysis, the defect density (*N_trp_*) of the synthesized SCs can be calculated with the following equation:

(2) $$N_{trap}=2\varepsilon \varepsilon_{0}V_{FTL}/(ed^{2})$$

where ε is the relative dielectric constant of CsPbBr_3_ (≈22), $\varepsilon_{0}$ is the vacuum dielectric constant, e is the electron charge, and d is the thickness of the as-prepared SCs (dsample1 ≈ 0.08 cm; dsample2 ≈ 0.10 cm). The *N_tr__a__p_* of the control CsPbBr_3_ SCs has been calculated to be 5.0 × 10^10^ cm^−3^ (Figure 2c), similar to previous reports. However, this has been reduced by an order in PEG-regulated CsPbBr_3_ SCs (5.5 × 10^9^ cm^−3^, Figure 2d), among the lowest trap densities reported to date (Table S1).

**Figure 3:**
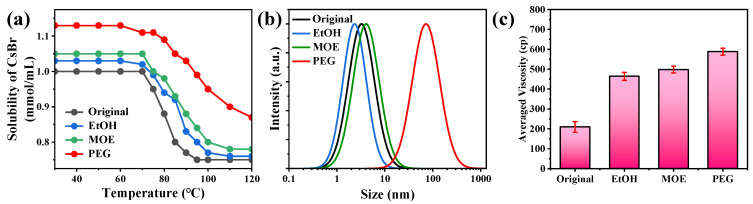
(**a**) The saturation solubility curves of the CsBr precursors in different solutions with temperature variations. (**b**) The dynamic light scattering (DLS) curves of the growth solution with different additives. (**c**) The viscosity of the control and additive-regulated precursors.

According to previous research, the poor solubility of CsBr is responsible for the blast nucleation of the ITC-grown CsPbBr_3_ SCs [35]. Figure 3a suggests that the solubility of CsBr can be greatly enhanced after adding PEG additives. Thus, the blast nucleation of the precursors at high temperatures can be largely eliminated. In addition, the PEG additive promotes the formation of large-sized crystal nuclei (Figure 3b and Figure S5), which is also beneficial for the growth of large SCs. According to Figure 3c, the viscosity of the precursors has been improved, which slows down the precipitation rate of additive-regulated precursors.

**Figure 4:**
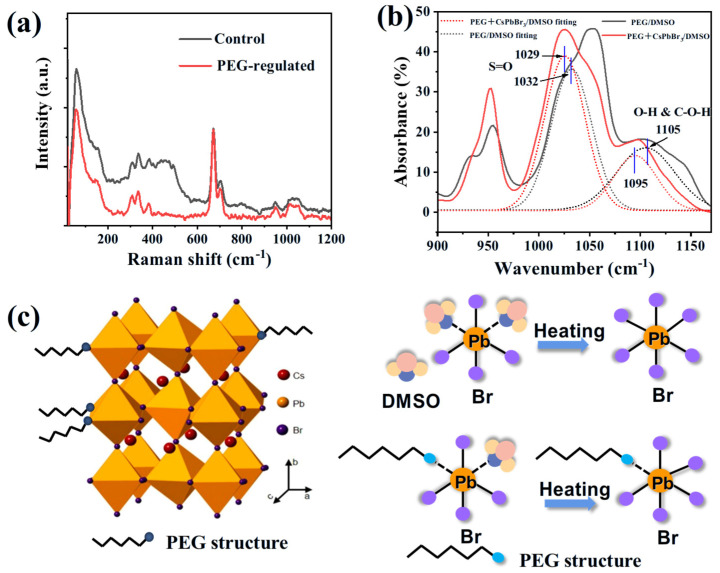
(**a**) Raman spectrum of the control and PEG—regulated CsPbBr_3_ precursor solution. The signal has been normalized according to the peak located at ~700 cm^−1^ (Pb-Br interaction). (**b**) Fourier transform infrared spectrometer (FTIR) analysis of the PEG dissolved in DMSO and PEG with CsPbBr_3_ DMSO solution. (**c**) Graphical illustration of the PEG-regulated CsPbBr_3_ SC growth.

Raman and Fourier transform infrared spectrometer (FTIR) analysis has been used to clarify the role of PEG-assisted growth. Raman analysis has been performed by exciting the precursor solution with a 532 nm laser at room temperature. The peak at low wavelength can be assigned to the Pb-Br bond [36]. This peak has been reduced after introducing PEG, indicating the stabilization of the Pb-Br bond after introducing the PEG additive. Figure 4b records the FTIR data of the precursor solutions. The peak at a wavenumber of 1032 cm^−1^, which belongs to the S=O bond of DMSO, has been shifted to 1029 cm^−1^ after introducing CsPbBr_3_ precursors. This downshifting tendency can be explained by the interaction between Pb^2+^ and the S=O bond [37]. A similar down-shifting tendency has been detected for the FTIR peak near 1105 cm^−1^. According to previous research, this peak is due to the stretching of the O-H and C-O-H bond [38]. This down-shifting can be explained as follows: the electrons have been dragged from the Pb^2+^ ions to the OH- species, which weakens the C-O bond and in turn, shifts the vibration frequency towards low wavenumbers [39]. The strong interaction between the OH- species and the Pb^2+^ can also be confirmed by XPS analysis (Figure S6).

**Figure 5:**
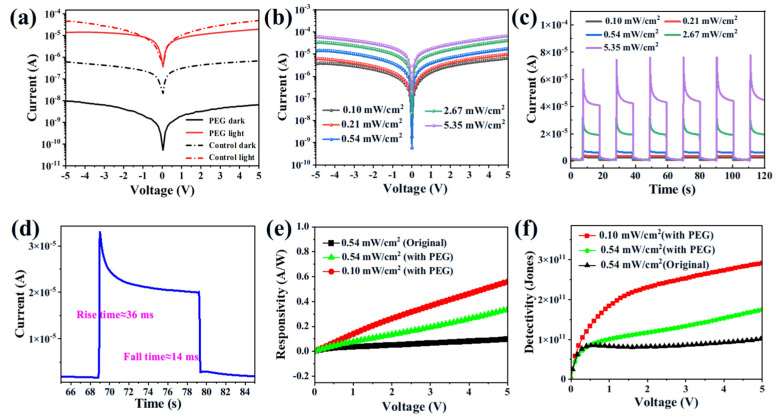
(**a**) Dark and light current—voltage (J—V) characteristics of the control and PEG—regulated CsPbBr_3_ device, and a 405 laser with a light intensity of 0.54 mW/cm^2^ has been used. (**b**) Light-dependent J—V characteristics of the PEG—regulated CsPbBr_3_ device, and the light intensity has been varied from 0.10 mW/cm^2^ to 5.35 mW/cm^2^. (**c**) The on—off current characteristics of the PEG-regulated CsPbBr_3_ device with different light intensities. (**d**) The response curve of the PEG—regulated CsPbBr_3_ device with 2.67 mW/cm^2^ illumination and a bias of 5V. The responsivity (**e**) and detectivity (**f**) curves of the two selective SC photodetector devices.

To investigate the potential of the as-grown SCs for photodetector applications, a planar-structured photodetector has been used (Figure S7). A 50 nm gold electrode has been deposited on the surface of the CsPbBr_3_ SCs with thermal evaporation. The distance of the electrode has been set to 75 um using a pre-designed mask. The devices have been measured by a two-probe station equipped with a wavelength and power-tuned light source. The dark current of PEG-regulated devices is two orders of magnitude lower than that of the control devices (Figure 5a), highlighting the low defect densities of the PEG-regulated SCs. After light illumination, under a bias of 5 V, the current of the PEG-regulated SCs has jumped from 6.46 × 10^−9^ A (dark) to 1.94 × 10^−5^ A (light illumination), corresponding to an on–off ratio of 3003. This on–off ratio is much higher than that of the control device (73), having approximately 40 times of enhancement. In addition, the current increases steadily with the increased light intensity (Figure 5b). The chopped transient photocurrent response has been listed in Figure 5c,d. The device exhibits remarkable operational stability under repeated on/off cycles. A current spike has been detected right after the lamp has been turned on, possibly due to the ion migration. After that, the incident current decreases until the mobile ions reach the equilibrium point (stabilized current) [28,38]. The rise time and fall times of the PEG-regulated photodetector during one chopped cycle have been calculated to be 36 ms and 14 ms, pointing to the fast response of our devices. Finally, taking the responsivity (R) and detectivity (D) into consideration, R can be defined as follows:

(3) $$R=\frac{I_{P}-I_{Dark}}{P}$$

where $I_{P}$ is photocurrent, $I_{Dark}$ is dark current, and P is the intensity of the incident light. The D can be calculated by the following equation:

(4) $$D=\frac{R}{\sqrt{\frac{2eI_{Dark}}{S}}}$$

Here, e is the elementary charge and S is the effective area of the electrode.

With a light intensity of 0.54 mA/cm^2^ and 5 V bias, the R has been calculated to be 0.37 A/W of the control SCs. This has been increased to 1.26 A/W in PEG-regulated SCs under the same condition (Figure 5e). The R of the PEG-regulated SCs has been further enhanced to 2.25 A/W with weak light illumination (0.1 mW/cm^2^). This indicates the potential of PEG-regulated SCs for weak light detection. As for D, this has been increased from 2.02 × 10^11^ Jones (control) to 3.39 × 10^11^ Jones (PEG-regulated). A weak light also promotes the D towards 6.07 × 10^11^ Jones in PEG-regulated SCs (Figure 5f). Listed in Table S2, the measured R and D are among the highest values reported to date, highlighting the good quality of our synthesized PEG-regulated SCs. As for the stability concern, the photocurrent of our PEG-regulated SCs has decreased by only 5.8% after being kept in ambient conditions with a relative humidity of 60% for two months (Figure S8). This superior stability can be attributed to the suppressed ion migration of the perovskite SCs [40,41,42].

## 3. Experiments

### 3.1. Material

Dimethyl sulfoxide (DMSO, AR) was purchased from Hushi chemicals (Shanghai, China). Anhydrous ethanol (EtOH, AR) was ordered from Fuyu Reagent (Tianjin, China). Lead bromide (PbBr_2_, >99.0%), cesium bromide (CsBr, >99.5%), and 2-Methoxyethanol (MOE, >99.5%) were purchased from Macklin (Shanghai, China). Polyethylene glycol (PEG—2000) was bought from Aladdin (Shanghai, China). All reagents and materials were used directly without further purification.

### 3.2. Growth of CsPbBr_3_ SC

The CsPbBr_3_ SCs used in this study were grown using the seed-assisted ITC method. Firstly, the growth solution was prepared by dissolving 2 mmol PbBr_2_ and 1 mmol CsBr in 2 mL DMSO. The solution was first stirred at 70 °C until completely dissolution. Subsequently, 0.1 mL of ethanol, 0.1 mL of 2-Methoxyethanol, and 0.05–0.1 g of PEG-2000 were added to the precursor solutions separately. The precursor solution was filtered with a 0.22 um filter and transferred to a clean vial. After heating the precursor solution at 110 °C for sufficient time, the CsPbBr_3_ SCs nucleated and grew with the evaporation of the DMSO solvent. In terms of the seed-assisted growth, tiny CsPbBr_3_ nuclei have been received from the supersaturated precursor solutions and transferred to another growth solution as the seed crystals. The obtained CsPbBr_3_ SCs were washed with hot DMF and dried in a vacuum drying oven for storage. All experiments were performed in ambient conditions.

### 3.3. Characterization

The structure of the as-grown CsPbBr_3_ SCs were characterized by the X-ray diffraction (XRD; Bruker, Karlsruhe, Germany). To investigate the optoelectronic properties of the synthesized CsPbBr_3_ SCs, the steady-state PL spectrum has been measured by Edinburgh FLS980 (Edinburgh Instruments Ltd., Livingston, UK)with an excitation wavelength of 400 nm. The TRPL measurements have been performed with Edinburgh FL980 using an excitation wavelength of 405 nm. The absorption spectra were collected by the UV-VIS spectrophotometer (Agilent Technologies, carey-8454; Agilent Technologies, Santa Clara, CA, USA). Au electrode has been deposited on the CsPbBr_3_ SCs with thermal evaporation. The viscosity of the precursor has been measured with a rotational rheometer (Anton Paar MCR92; Anton Paar GmbH, Graz, Austria) at 25 °C. The shear rate range has been set at 0.1–1000 s^−1^. Five datasets have been collected and the results have been recorded in Figure 3c. The shape of the electrode has been controlled by a pre-designed mask. The current–voltage (J-V) curves of the Au/CsPbBr_3_/Au-structured photodetectors were recorded with the Keithley 2400 (Solon, OH, USA) source measurement unit.

## 4. Summary

In summary, we introduce an additive-tuning strategy to solve the blast nucleation of ITC-grown CsPbBr_3_ SCs. First and foremost, the oxhydryl groups on PEG bond strongly with the PbBr_n_^(n−2)−^ clusters, which reduces the number of nucleation species during crystal growth. In addition, the high viscosity of the PEG additive is also beneficial for the smooth growth of the SCs, resulting in defectless crystals. Finally, our PEG-regulated CsPbBr_3_ SCs show good responsivity (2.25 A/W) and detectivity (6.07 × 10^11^ Jones) to weak light, demonstrating their significant potential in the field of optoelectronic devices.

## Data Availability

Data are contained within the article and Supplementary Materials. Further inquiries can be directed to the corresponding author.

## Supplementary Materials

The following supporting information can be downloaded at https://www.mdpi.com/article/10.3390/molecules30224423/s1; Figure S1: The influence of PEG concentration on the quality of the ITC-grown CsPbBr_3_ SCs; Figure S2: Photographs of the as-synthesized CsPbBr_3_ SCs; Figure S3: Surface-view SEM images of the control, EtOH, MOE, and PEG-regulated CsPbBr_3_ SCs; Figure S4: Atomic force microscope (AFM) characterization of the control (a–c) and PEG-regulated CsPbBr_3_ SCs (d–f). a, d. three-dimensional surface images; b, e. two-dimensional surface images; c, f. Height profile; Figure S5: Side-view and bottom-view of the mother solution with 1.1 mmol/mL CsBr precursors; Figure S6: X-ray photoelectron spectroscopy (XPS) Pb 4f orbital curve of the control and PEG-regulated CsPbBr_3_ powder; Figure S7: Photographs and device structure of the as-prepared photodetectors; Figure S8: Stability evaluation of the PEG-regulated SC devices after a two-month storage; Table S1: The defect state concentration of CsPbBr_3_ SCs reported by different researchers; Table S2: The summarized responsivity (R) and detectivity (D) values of the CsPbBr3 SCs for photodetection applications. Refs. [20,43,44,45,46,47,48,49,50,51,52,53,54,55,56] are cited in Supplementary Materials.

## References

[1] Miyata K., Zhu X.Y. (2018). Ferroelectric large polarons. Nat. Mater..

[2] Zhao B., Zhang T., Liu C., Li Z., Liu W., Bai Y., Wang T., Sun X., Zhu S., Chen Y. (2022). Hydroxyl substituted Spiro-OMeTAD as multi-site defect healing and carrier extraction enhanced surface passivator toward efficient perovskite solar cells. Mater. Today Energy.

[3] Veldhuis S.A., Boix P.P., Yantara N., Li M., Sum T.C., Mathews N., Mhaisalkar S.G. (2016). Perovskite materials for light-emitting diodes and lasers. Adv. Mater..

[4] Sun J., Ding L. (2023). Linearly Polarization-Sensitive Perovskite Photodetectors. Nano-Micro Lett..

[5] Li Z., Zhou F., Yao H., Ci Z., Yang Z., Jin Z. (2021). Halide perovskites for high-performance X-ray detector. Mater. Today.

[6] Chen Y., He M., Peng J., Sun Y., Liang Z. (2016). Structure and growth control of organic–inorganic halide perovskites for optoelectronics: From polycrystalline films to single crystals. Adv. Sci..

[7] Tan Q., Li Z., Luo G., Zhang X., Che B., Chen G., Gao H., He D., Ma G., Wang J. (2023). Inverted perovskite solar cells using dimethylacridine-based dopants. Nature.

[8] Zhang T., Zhao B., Li Z., Liu S., Liu C., Li X., Liu H., Chen Y., Liu Z., Li X. (2022). Inspired from Spiro-OMeTAD: Developing ambipolar spirobifluorene derivatives as effective passivation molecules for perovskite solar cells. J. Mater. Chem. C.

[9] Lin K., Xing J., Quan L.N., de Arquer F.P.G., Gong X., Lu J., Xie L., Zhao W., Zhang D., Yan C. (2018). Perovskite light-emitting diodes with external quantum efficiency exceeding 20 per cent. Nature.

[10] Ma D., Lin K., Dong Y., Choubisa H., Proppe A.H., Wu D., Wang Y.-K., Chen B., Li P., Fan J.Z. (2021). Distribution control enables efficient reduced-dimensional perovskite LEDs. Nature.

[11] Pan L., Shrestha S., Taylor N., Nie W., Cao L.R. (2021). Determination of X-ray detection limit and applications in perovskite X-ray detectors. Nat. Commun..

[12] Massuyeau F., Broux T., Coulet F., Demessence A., Mesbah A., Gautier R. (2022). Perovskite or Not Perovskite? A Deep-Learning Approach to Automatically Identify New Hybrid Perovskites from X-ray Diffraction Patterns. Adv. Mater..

[13] He Y., Stoumpos C.C., Hadar I., Luo Z., McCall K.M., Liu Z., Chung D.Y., Wessels B.W., Kanatzidis M.G. (2021). Demonstration of energy-resolved γ-ray detection at room temperature by the CsPbCl3 perovskite semiconductor. J. Am. Chem. Soc..

[14] Cao G., Zhang H., Wang C., Li X. (2022). Self-Driving Perovskite Dember Photodetectors. Adv. Opt. Mater..

[15] Hou H.Y., Tian S., Ge H.R., Chen J.D., Li Y.Q., Tang J.X. (2022). Recent progress of polarization-sensitive perovskite photodetectors. Adv. Funct. Mater..

[16] Rao H.S., Li W.G., Chen B.X., Kuang D.B., Su C.Y. (2017). In situ growth of 120 cm2 CH3NH3PbBr3 perovskite crystal film on FTO glass for narrowband-photodetectors. Adv. Mater..

[17] Maculan G., Sheikh A.D., Abdelhady A.L., Saidaminov M.I., Haque M.A., Murali B., Alarousu E., Mohammed O.F., Wu T., Bakr O.M. (2015). CH_3_NH_3_PbCl_3_ single crystals: Inverse temperature crystallization and visible-blind UV-photodetector. J. Phys. Chem. Lett..

[18] Xue J., Yang D., Cai B., Xu X., Wang J., Ma H., Yu X., Yuan G., Zou Y., Song J. (2019). Photon-induced reversible phase transition in CsPbBr_3_ perovskite. Adv. Funct. Mater..

[19] Yu J., Liu G., Chen C., Li Y., Xu M., Wang T., Zhao G., Zhang L. (2020). Perovskite CsPbBr_3_ crystals: Growth and applications. J. Mater. Chem. C.

[20] Zhao X., Wang S., Zhuge F., Zhu N., Song Y., Fu M., Deng Z., Fang X., Meng G. (2023). Nucleation-controlled growth of high-quality CsPbBr_3_ single crystals for ultrasensitive weak-light photodetectors. J. Mater. Chem. C.

[21] Zheng W., Lin R., Zhang Z., Huang F. (2018). Vacuum-ultraviolet photodetection in few-layered h-BN. ACS Appl. Mater. Interfaces.

[22] Raja S.N., Bekenstein Y., Koc M.A., Fischer S., Zhang D., Lin L., Ritchie R.O., Yang P., Alivisatos A.P. (2016). Encapsulation of Perovskite Nanocrystals into Macroscale Polymer Matrices: Enhanced Stability and Polarization. ACS Appl. Mater. Interfaces.

[23] Konidakis I., Karagiannaki A., Stratakis E. (2022). Advanced composite glasses with metallic, perovskite, and two-dimensional nanocrystals for optoelectronic and photonic applications. Nanoscale.

[24] Liu Y., Zheng X., Fang Y., Zhou Y., Ni Z., Xiao X., Chen S., Huang J. (2021). Ligand assisted growth of perovskite single crystals with low defect density. Nat. Commun..

[25] Wang H., Wang X., Chen R., Zhang H., Wang X., Wang J., Zhang J., Mu L., Wu K., Fan F. (2018). Promoting photocatalytic H_2_ evolution on organic–inorganic hybrid perovskite nanocrystals by simultaneous dual-charge transportation modulation. ACS Energy Lett..

[26] Song J., Cui Q., Li J., Xu J., Wang Y., Xu L., Xue J., Dong Y., Tian T., Sun H. (2017). Ultralarge all-inorganic perovskite bulk single crystal for high-performance visible–infrared dual-modal photodetectors. Adv. Opt. Mater..

[27] He Y., Matei L., Jung H.J., McCall K.M., Chen M., Stoumpos C.C., Liu Z., Peters J.A., Chung D.Y., Wessels B.W. (2018). High spectral resolution of gamma-rays at room temperature by perovskite CsPbBr_3_ single crystals. Nat. Commun..

[28] Wang F., Bai R., Sun Q., Liu X., Cheng Y., Xi S., Zhang B., Zhu M., Jiang S., Jie W. (2022). Precursor engineering for solution method-grown spectroscopy-grade CsPbBr_3_ crystals with high energy resolution. Chem. Mater..

[29] Liu Z., Peters J.A., Pan L., Klepov V., De Siena M., Benadia A., Chung D.Y., Kanatzidis M.G., Wessels B.W. (2023). Investigation of defects in melt and solution grown perovskite CsPbBr_3_ single crystals. Appl. Phys. Lett..

[30] Dirin D.N., Cherniukh I., Yakunin S., Shynkarenko Y., Kovalenko M.V. (2016). Solution-grown CsPbBr_3_ perovskite single crystals for photon detection. Chem. Mater..

[31] Cheng Y., Zhu M., Wang F., Bai R., Yao J., Jie W., Xu Y. (2021). Precursor solution-dependent secondary phase defects in CsPbBr_3_ single crystal grown by inverse temperature crystallization. J. Mater. Chem. A.

[32] Feng Y., Pan L., Wei H., Liu Y., Ni Z., Zhao J., Rudd P.N., Cao L.R., Huang J. (2020). Low defects density CsPbBr_3_ single crystals grown by an additive assisted method for gamma-ray detection. J. Mater. Chem. C.

[33] Zhang H., Liu X., Dong J., Yu H., Zhou C., Zhang B., Xu Y., Jie W. (2017). Centimeter-Sized Inorganic Lead Halide Perovskite CsPbBr_3_ Crystals Grown by an Improved Solution Method. Cryst. Growth Des..

[34] Stoumpos C.C., Malliakas C.D., Peters J.A., Liu Z., Sebastian M., Im J., Chasapis T.C., Wibowo A.C., Chung D.Y., Freeman A.J. (2013). Crystal growth of the perovskite semiconductor CsPbBr_3_: A new material for high-energy radiation detection. Cryst. Growth Des..

[35] Wei X., Liu H., Zhang Z., Xu W., Huang W., Luo L.-B., Liu J. (2021). Low-temperature architecture of a cubic-phase CsPbBr_3_ single crystal for ultrasensitive weak-light photodetectors. Chem. Commun..

[36] Ma L., Yan Z., Zhou X., Pi Y., Du Y., Huang J., Wang K., Wu K., Zhuang C., Han X. (2021). A polymer controlled nucleation route towards the generalized growth of organic-inorganic perovskite single crystals. Nat. Commun..

[37] Zhang T., Dar M.I., Li G., Xu F., Guo N., Grätzel M., Zhao Y. (2017). Bication lead iodide 2D perovskite component to stabilize inorganic α-CsPbI_3_ perovskite phase for high-efficiency solar cells. Sci. Adv..

[38] Shameli K., Ahmad M.B., Jazayeri S.D., Sedaghat S., Shabanzadeh P., Jahangirian H., Mahdavi M., Abdollahi Y. (2012). Synthesis and characterization of polyethylene glycol mediated silver nanoparticles by the green method. Int. J. Mol. Sci..

[39] Liu D., Hu Z., Hu W., Wangyang P., Yu K., Wen M., Zu Z., Liu J., Wang M., Chen W. (2017). Two-step method for preparing all-inorganic CsPbBr3 perovskite film and its photoelectric detection application. Mater. Lett..

[40] Zhang Y., Liu Y., Xu Z., Yang Z., Liu S. (2020). 2D Perovskite Single Crystals with Suppressed Ion Migration for High-Performance Planar-Type Photodetectors. Small.

[41] Huang J., Zhang H., Zhu H., Zhang C., Chen M., Cao D. (2025). Buried Interfacial Engineering with Potassium Hypophosphite to Suppress Ion Migration for Improved and Stabilized Perovskite Photodetectors. ACS Appl. Electron. Mater..

[42] Hua Y., Zhang G., Sun X., Zhang P., Hao Y., Xu Y., Yang Y., Lin Q., Li X., Zhai Z. (2024). Suppressed ion migration for high-performance X-ray detectors based on atmosphere-controlled EFG-grown perovskite CsPbBr_3_ single crystals. Nat. Photonics.

[43] Zhang P., Zhang G., Liu L., Ju D., Zhang L., Cheng K., Tao X. (2018). Anisotropic Optoelectronic Properties of Melt-Grown Bulk CsPbBr_3_ Single Crystal. J. Phys. Chem. Lett.

[44] Zhang P., Hua Y., Xu Y., Sun Q., Li X., Cui F., Liu L., Bi Y., Zhang G., Tao X. (2022). Ultrasensitive and Robust 120 keV Hard X-Ray Imaging Detector based on Mixed-Halide Perovskite CsPbBr_3_−I Single Crystals. Adv. Mater.

[45] Miao X., Qiu T., Zhang S., Ma H., Hu Y., Bai F., Wu Z. (2017). Air-stable CsPb_1−x_Bi_x_Br_3_ (0 ≤ x ≪ 1) Perovskite Crystals: Optoelectronic and Photostriction Properties. J. Mater. Chem. C.

[46] Fan Z., Liu J., Zuo W., Liu G., He X., Luo K., Ye Q., Liao C. (2020). Solution-Processed MAPbBr_3_ and CsPbBr_3_ Single-Crystal Detectors with Improved X-Ray Sensitivity via Interfacial Engineering. Phys. Status Solidi A.

[47] Zhao C., Tian W., Liu J., Sun Q., Luo J., Yuan H., Gai B., Tang J., Guo J., Jin S. (2019). Stable Two-Photon Pumped Amplified Spontaneous Emission from Millimeter-Sized CsPbBr_3_ Single Crystals. J. Phys. Chem. Lett.

[48] Peng J., Xia C.Q., Xu Y., Li R., Cui L., Clegg J.K., Herz L.M., Johnston M.B., Lin Q. (2021). Crystallization of CsPbBr_3_ Single Crystals in Water for X-ray Detection. Nat. Commun.

[49] Gao L., Sun J.L., Li Q., Yan Q. (2022). γ-ray Radiation Hardness of CsPbBr_3_ Single Crystals and Single-Carrier Devices. ACS Appl. Mater. Interfaces.

[50] Wang K., Jing L., Yao Q., Zhang J., Cheng X., Yuan Y., Shang C., Ding J., Zhou T., Sun H. (2021). Highly In-Plane Polarization-Sensitive Photodetection in CsPbBr_3_ Single Crystal. J. Phys. Chem. Lett.

[51] Ding J., Du S., Zuo Z., Zhao Y., Cui H., Zhan X. (2017). High Detectivity and Rapid Response in Perovskite CsPbBr_3_ Single-Crystal Photodetector. J. Phys. Chem. C.

[52] Saidaminov M.I., Haque M.A., Almutlaq J., Sarmah S., Miao X.H., Begum R., Zhumekenov A.A., Dursun I., Cho N., Murali B. (2017). Inorganic Lead Halide Perovskite Single Crystals: Phase-Selective Low-Temperature Growth, Carrier Transport Properties, and Self-Powered Photodetection. Adv. Opt. Mater.

[53] Cai J., Zhao T., Chen M., Su J., Shen X., Liu Y., Cao D. (2022). Ion Migration in the All-Inorganic Perovskite CsPbBr_3_ and Its Impacts on Photodetection. J. Phys. Chem. C.

[54] Cha J.H., Han J.H., Yin W., Park C., Park Y., Ahn T.K., Cho J.H., Jung D.Y. (2017). Photoresponse of CsPbBr_3_ and Cs_4_PbBr_6_ Perovskite Single Crystals. J. Phys. Chem. Lett.

[55] Yuan Y., Chen M., Yang S., Shen X., Liu Y., Cao D., Xing G., Tang Z. (2022). Improved CsPbBr_3_ Visible Light Photodetectors via Decoration of Sputtered Au Nanoparticles with Synergistic Benefits. Nano Select.

[56] Cheng P., Liu Z., Kang R., Zhou J., Wang X., Zhao J., Zuo Z. (2023). Growth and High-Performance Photodetectors of CsPbBr_3_ Single Crystals. ACS OMEGA.

