# Supplementary material for: Blast Nucleation Suppressed Growth of Large-Sized High-Quality CsPbBr3 Single Crystals for Photodetector Applications
Source: Molecules. 2025 Nov 16;30(22):4423. doi: 10.3390/molecules30224423 (PMC12655495; doi:10.3390/molecules30224423)
Supplement: Supplementary file 1 [file molecules-30-04423-s001.zip › molecules-3814712-supplementary.pdf]

Supporting Information for

Blast Nucleation Suppressed Growth of Large-Sized High-Quality $CsPbBr_3$ Single Crystals for Photodetector Applications

Xinyu Sun [1,†], Yuxia Yin [1,†], Xiaolin Xia [2] and Teng Zhang [1,*]

[1] Shan Dong Key Laboratory of Intelligent Energy Materials, School of Materials Science and Engineering, China University of Petroleum (EastChina), Qingdao 266580, China; upc_sxy9764@163.com (X.S.); z23140095@s.upc.edu.cn (Y.Y.)

[2] School of Information Science and Engineering, Qingdao Institute of Technology, Qingdao 266300, China; xiaxiaolin@qit.edu.cn

* Correspondence: tzhangae@connect.ust.hk

† These authors contributed equally to this work.

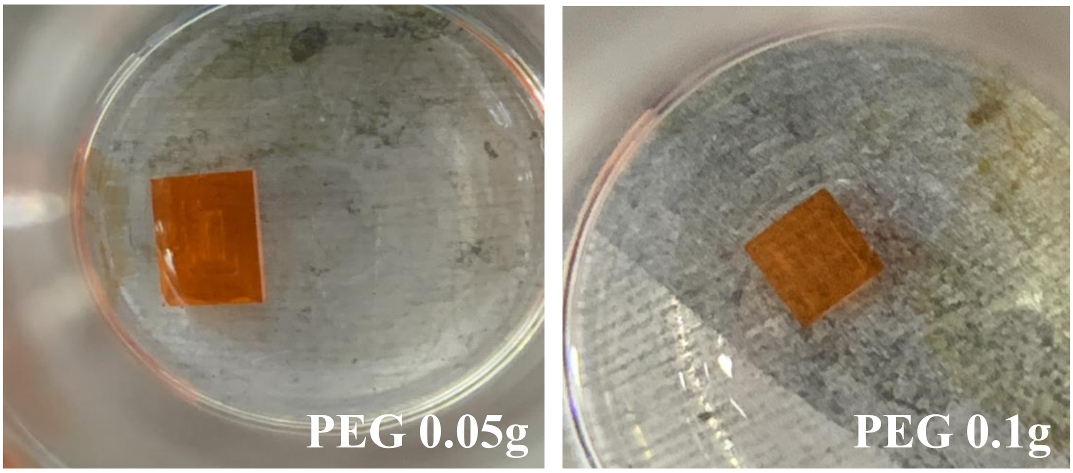

Figure S1 The influence of PEG concentration on the quality of the ITC grown $CsPbBr_3$ SCs.

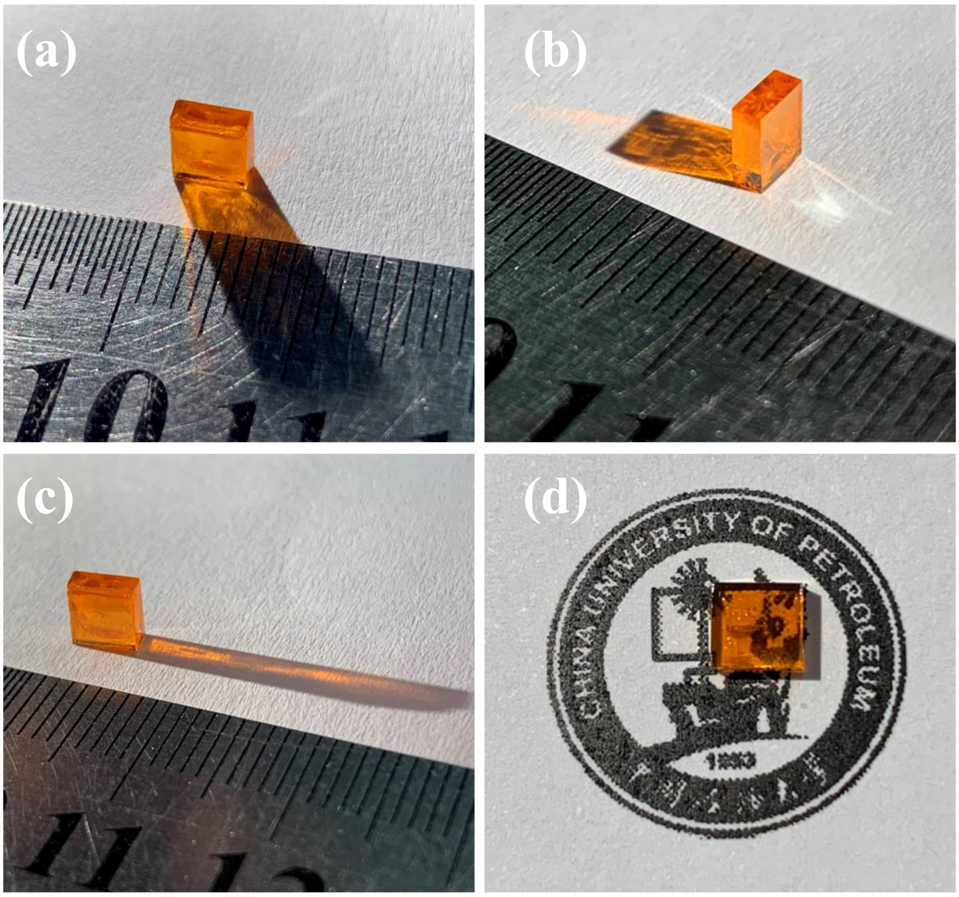

Figure S2 Photographs of the as-synthesized $CsPbBr_3$ SCs.

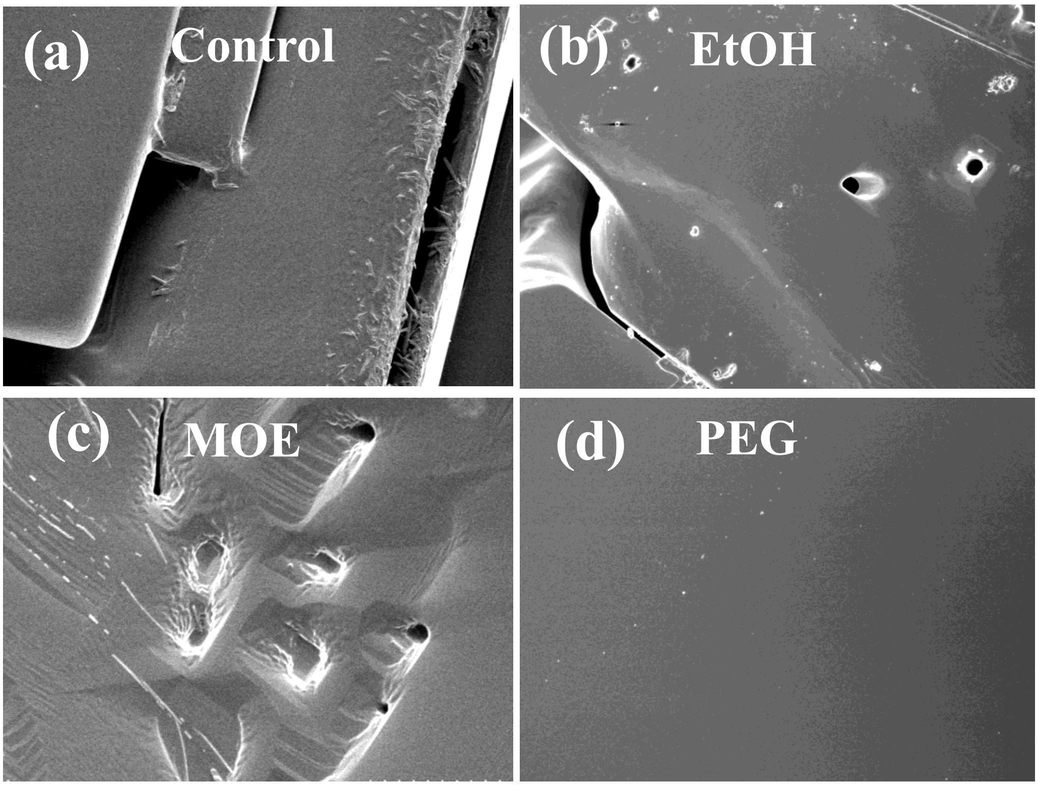

Figure S3 Surface-view SEM images of the control, EtOH, MOE and PEG-regulated $CsPbBr_3$ SCs.

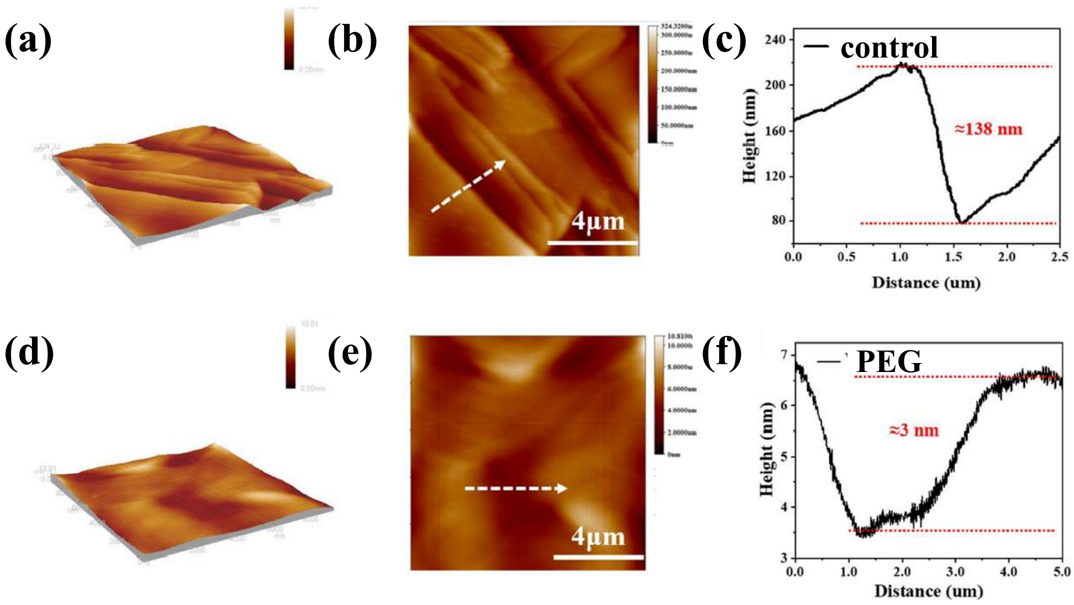

Figure S4 Atomic Force Microscope (AFM) characterization of the control (a-c) and PEG-regulated $CsPbBr_3$ SCs (d-f). a, d 3-dimensional surface images, b, e 2-dimensional surface images, c, f height profile.

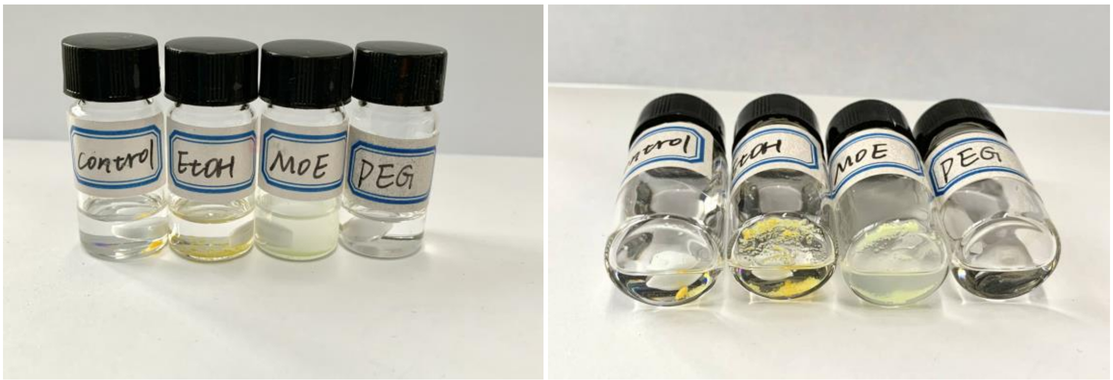

Figure S5 Side-view and bottom-view of the mother solution with 1.1 mmol/mL CsBr precursors.

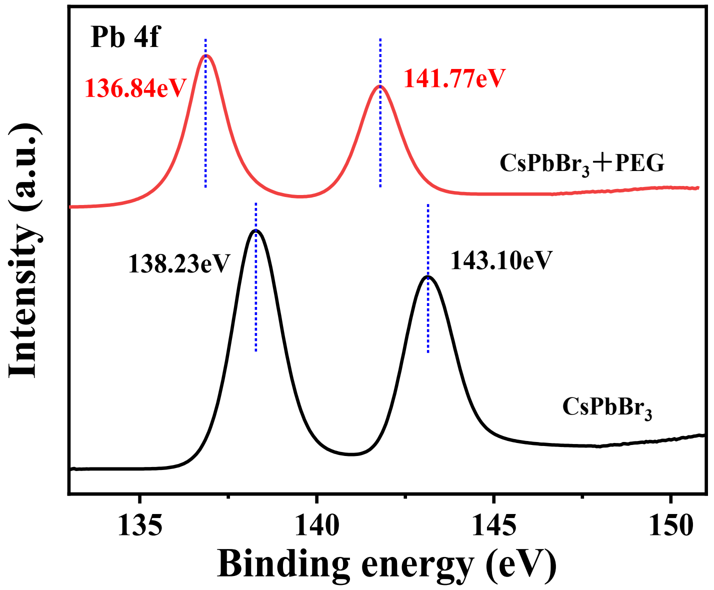

Figure S6 X-ray Photoelectron Spectroscopy (XPS) Pb 4f orbital curve of the control and PEG-regulated $CsPbBr_3$ powder.

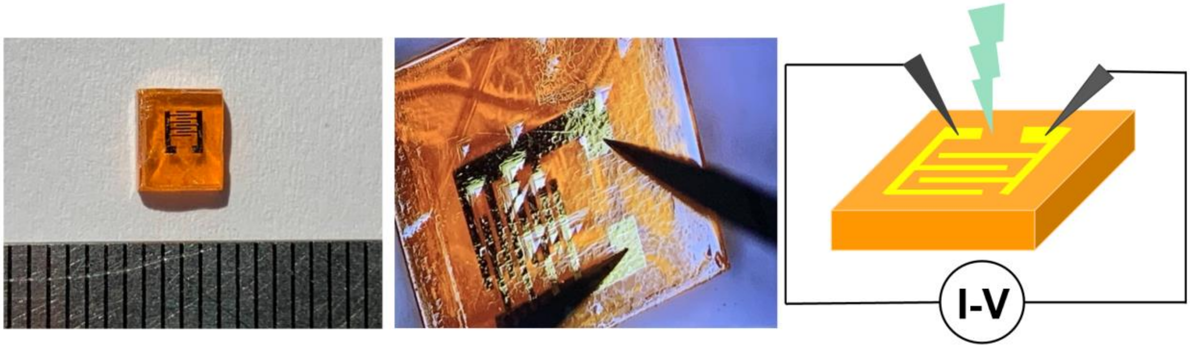

Figure S7 Photographs and device structure of the as-prepared photodetectors.

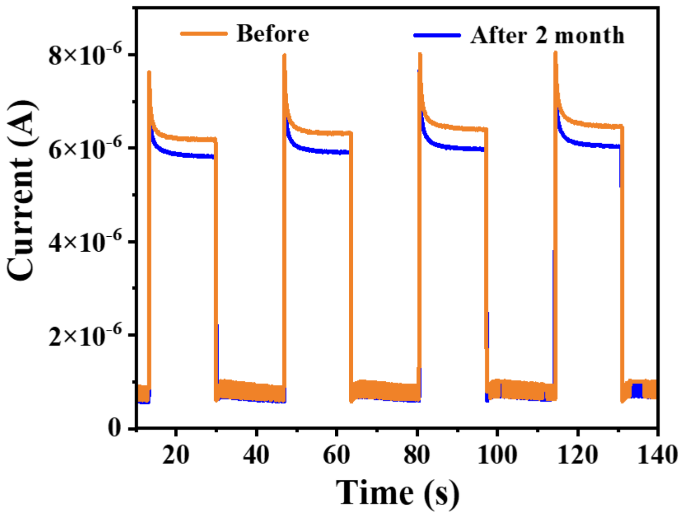

Figure S8 Stability evaluation of the PEG-regulated SC devices after a 2-month storage.

Table S1 The defect state concentration of $CsPbBr_3$ SCs reported by different researchers.

**Method**	**Ntrap (cm^{-3})**	**Reference**
Bridgman	1.08×10^{9}	1
Bridgman	3.03×10^{11}	2
AVC	2.80×10^{10}	3
AVC	3.87×10^{10}	4
AVC	8.50×10^{9}	5
STL	1.70×10^{10}	6
ITC	1.10×10^{10}	7
ITC	7.45×10^{9}	8
ITC	$5.05*10^{9}$	This work

Table S2 The summarized responsivity (R) and detectivity (D) values of the $CsPbBr_3$ SCs for photodetection applications.

Materials	R(A/W)	D(Jones)	on/off ratio	Rise/fall time (ms)	ref
$CsPbBr_3$ SC	-	-	228 (6 V)	-	8
$CsPbBr_3$ SC	0.028 (5 V)	1.8×10^{11}	100 (5 V)	57/46.8	9
$CsPbBr_3$ SC	0.028(5 V)	1.7×10^{11}	105 (0 V)	230/60	10
$CsPbBr_3$ SC	~ 4.25	~ 1.2×10^{11}	55 (3 V)	-	11
$CsPbBr_3$ SC	2.1	-	460 (6 V)	300/5000	12
$CsPbBr_3$ SC	0.088 (3 V)	-	122 (3 V)	48/53	13
$CsPbBr_3$ SC	0.063 (10 V)	2.06×10^{11}	1017 (10 V)	0.33	14
$CsPbBr_3$ SC	36.4 (10 V)	-	104	0.003/0.006	15
$CsPbBr_3$ SC	0.559 (5 V)	2.91×10^{11}	3003 (5V)	36/14	This work

Reference

1. Zhang, P.; Zhang, G.; Liu, L.; Ju, D.; Zhang, L.; Cheng, K.; Tao, X., Anisotropic Optoelectronic Properties of Melt-Grown Bulk $CsPbBr_3$ Single Crystal. *J. Phys. Chem. Lett* **2018**, *9* (17), 5040-5046.

2. Zhang, P.; Hua, Y.; Xu, Y.; Sun, Q.; Li, X.; Cui, F.; Liu, L.; Bi, Y.; Zhang, G.; Tao, X., Ultrasensitive and Robust 120 keV Hard X-Ray Imaging Detector based on Mixed-Halide Perovskite $CsPbBr_{3-n}I_n$ Single Crystals. *Adv. Mater* **2022**, *34* (12), 2106562.

3. Miao, X.; Qiu, T.; Zhang, S.; Ma, H.; Hu, Y.; Bai, F.; Wu, Z., Air-stable $CsPb_{1-x}Bi_xBr_3$ ($0 \le x \ll 1$) Perovskite Crystals: Optoelectronic and Photostriction Properties. *J. Mater. Chem. C* **2017**, *5* (20), 4931-4939.

4. Fan, Z.; Liu, J.; Zuo, W.; Liu, G.; He, X.; Luo, K.; Ye, Q.; Liao, C., Solution-Processed $MAPbBr_3$ and $CsPbBr_3$ Single-Crystal Detectors with Improved X-Ray Sensitivity via Interfacial Engineering. *Phys. Status Solidi A* **2020**, *217* (9), 2000104.

5. Zhao, C.; Tian, W.; Liu, J.; Sun, Q.; Luo, J.; Yuan, H.; Gai, B.; Tang, J.; Guo, J.; Jin, S., Stable Two-Photon Pumped Amplified Spontaneous Emission from Millimeter-Sized $CsPbBr_3$ Single Crystals. *J. Phys. Chem. Lett* **2019**, *10* (10), 2357-2362.

6. Peng, J.; Xia, C. Q.; Xu, Y.; Li, R.; Cui, L.; Clegg, J. K.; Herz, L. M.; Johnston, M. B.; Lin, Q., Crystallization of $CsPbBr_3$ Single Crystals in Water for X-ray Detection. *Nat. Commun* **2021**, *12* (1), 1531.

7. Gao, L.; Sun, J. L.; Li, Q.; Yan, Q., γ-ray Radiation Hardness of $CsPbBr_3$ Single Crystals and Single-Carrier Devices. *ACS Appl. Mater. Interfaces* **2022**, *14* (33), 37904-37915.

8. Wang, K.; Jing, L.; Yao, Q.; Zhang, J.; Cheng, X.; Yuan, Y.; Shang, C.; Ding, J.; Zhou, T.; Sun, H.; Zhang, W.; Li, H., Highly In-Plane Polarization-Sensitive Photodetection in $CsPbBr_3$ Single Crystal. *J. Phys. Chem. Lett* **2021**, *12* (7), 1904-1910.

9. Ding, J.; Du, S.; Zuo, Z.; Zhao, Y.; Cui, H.; Zhan, X., High Detectivity and Rapid Response in Perovskite $CsPbBr_3$ Single-Crystal Photodetector. *J. Phys. Chem. C* **2017**, *121* (9), 4917-4923.

10. Saidaminov, M. I.; Haque, M. A.; Almutlaq, J.; Sarmah, S.; Miao, X. H.; Begum, R.; Zhumekenov, A. A.; Dursun, I.; Cho, N.; Murali, B.; Mohammed, O. F.; Wu, T.; Bakr, O. M., Inorganic Lead Halide Perovskite Single Crystals: Phase-Selective Low-Temperature Growth, Carrier Transport Properties, and Self-Powered Photodetection. *Adv. Opt. Mater* **2017**, *5* (2), 1600704.

11. Cai, J.; Zhao, T.; Chen, M.; Su, J.; Shen, X.; Liu, Y.; Cao, D., Ion Migration in the All-Inorganic Perovskite $CsPbBr_3$ and Its Impacts on Photodetection. *J. Phys. Chem. C* **2022**, *126* (23), 10007-10013.

12. Cha, J. H.; Han, J. H.; Yin, W.; Park, C.; Park, Y.; Ahn, T. K.; Cho, J. H.; Jung, D. Y., Photoresponse of $CsPbBr_3$ and Cs_4PbBr_6 Perovskite Single Crystals. *J. Phys. Chem. Lett* **2017**, *8* (3), 565-570.

13. Yuan, Y.; Chen, M.; Yang, S.; Shen, X.; Liu, Y.; Cao, D.; Xing, G.; Tang, Z., Improved $CsPbBr_3$ Visible Light Photodetectors via Decoration of Sputtered Au Nanoparticles with Synergistic Benefits. *Nano Select* **2022**, *3* (1), 178-187.

14. Cheng, P.; Liu, Z.; Kang, R.; Zhou, J.; Wang, X.; Zhao, J.; Zuo, Z., Growth and High-Performance Photodetectors of $CsPbBr_3$ Single Crystals. *ACS OMEGA* **2023**, *8* (29), 26351-26358.

15. Zhao, X.; Wang, S.; Zhuge, F.; Zhu, N.; Song, Y.; Fu, M.; Deng, Z.; Fang, X.; Meng, G., Nucleation-Controlled Growth of High-Quality $CsPbBr_3$ Single Crystals for Ultrasensitive Weak-Light Photodetectors. *J. Phys. Chem. C* **2023**, *11* (25), 8533-8540.
